# OnabotulinumtoxinA blockade of the sphenopalatine ganglion in treatment-resistant chronic migraine (MiBlock): rationale, design and study protocol for a multi-centre, investigator initiated, randomised, quadruple-blinded placebo-controlled trial.

**DOI:** 10.12688/f1000research.164241.1

**Published:** 2025-06-06

**Authors:** Tore Wergeland, Melanie Simpson, Irina Aschehoug, Lise Øie, Sozaburo Hara, Einar Tobias Vassbø Skalstad, Øyvind Salvesen, Erling Tronvik, Daniel Bratbak

**Affiliations:** 1 Norwegian Centre for Headache Research (NorHEAD), Norwegian University of Science and Technology, Trondheim, Norway; 2 Department of neurology and neurophysiology, St Olav's University Hospital, Trondheim, Norway; 3 Department of Neuromedicine and Movement Sciences, Norwegian University of Science and Technology, Trondheim, Norway; 4 Department of Public Health and Nursing, Norwegian University of Science and Technology, Trondheim, Norway; 5 Department of Neurosurgery, St Olav's University Hospital, Trondheim, Norway; 6 Faculty of medicine and health sciences, Norwegian University of Science and Technology, Trondheim, Norway

**Keywords:** chronic migraine, Sphenopalatine ganglion, migraine prevention

## Abstract

**Background:**

Treatment-resistant chronic migraine patients constitute a heavily burdened patient population with need for novel treatment options. The sphenopalatine ganglion (SPG), located deep within the facial structures, is a synaptic junction in the trigemino-autonomic reflex that is hypothesised to play a part in migraine pathophysiology. A 2017 open-label pilot study explored the SPG as a target for onabotulinumtoxinA injections in chronic migraine using a navigation-guided injection device, demonstrating a favourable safety profile and promising response on migraine related efficacy outcomes.

**Methods:**

MiBlock is a multi-centre, investigator-initiated, publicly funded, randomised, quadruple-blinded and placebo-controlled trial. Participation is voluntary and all participants must sign a written consent prior to inclusion. The study is approved by the Norwegian ethics committee and the Norwegian Medical Product Agency. Patients with treatment-resistant chronic migraine will be recruited from four Norwegian university hospitals until 170 have received study treatment. The study will investigate the efficacy of a single-session, bilateral, navigation-guided, percutaneous injection of either onabotulinumtoxinA (verum) or 0.9% NaCl (placebo) towards the SPG under local anaesthesia. The primary efficacy endpoint is the change from baseline in the frequency of moderate and severe headache days at weeks 5-8 post-injection. The primary efficacy outcome variable is collected prospectively through daily headache eDiary entries during the entire study participation. The treatment effect will be assessed by comparing the placebo and the treatment arm according to a prespecified statistical analysis plan.

**Discussion:**

This trial addresses a novel treatment strategy in headache prevention. The study is designed to evaluate the efficacy and safety of onabotulinumtoxinA injection towards the SPG in chronic migraine, but results may shed light on the feasibility of navigation-guided SPG injections in several headache disorders. Results will be published in international open-access peer-reviewed journals.

**Trial registration number:**

EudraCT number: 2018-004053-24.

ClinicalTrial ref: NCT04069897.

Protocol version 4.2, Date 21.11.2019

## Introduction

Migraine is a severe brain condition, listed as the sixth most disabling disorder by the World Health Organization, ^1, 2^ and the first cause of disability in those under 50 years of age. ^3^ In population-based studies, chronic migraine has a prevalence of 1.4-2.2%. ^4^ Preventive treatment strategies consist of oral medications, subcutaneous injections with botulinum toxin according to the PREEMPT-regimen and the recently introduced CGRP-targeting therapies. The latter are to date the only available preventives which development was clearly sparked by advances in insights of underlying migraine pathophysiology, and thus showcase the advantages of a mechanistic approach in novel therapy development. ^5^ Despite such recent advances, a substantial portion of patients remains insufficiently relieved warranting development of new treatment strategies.

**Figure 1.**
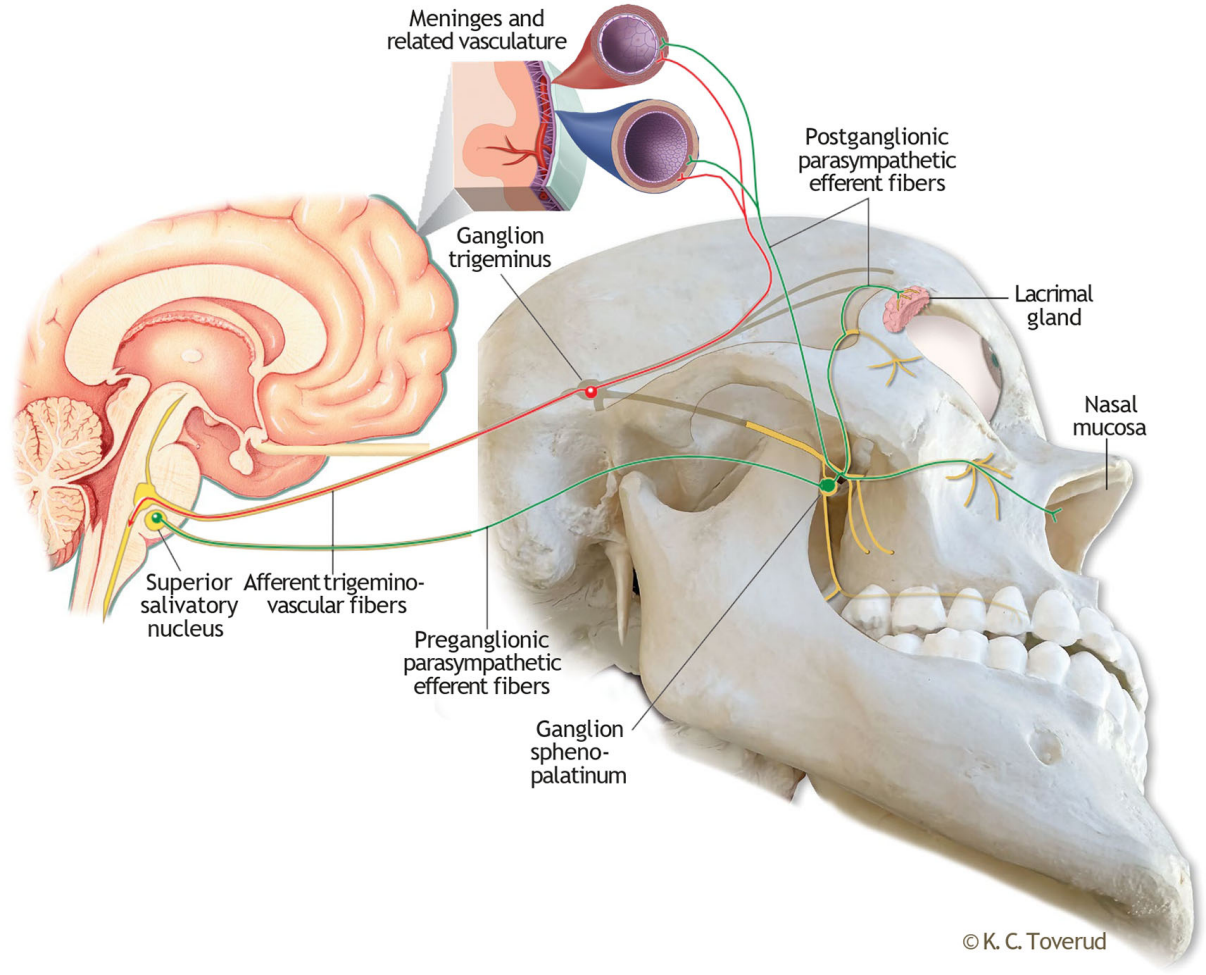
Illustration of the trigemino-autonomic brainstem reflex arc. The afferent part of this brainstem reflex (red) is the trigemino-vascular coupling, which for simplicity is illustrated with first branch meningeal innervation only. The efferent part (green) consists of parasympathetic fibers with a synaptic junction in the sphenopalatine ganglion. Postganglionic fibers proceed to the lacrimal gland, nasal mucosa and intracranially to the meninges and related vasculature. Enlarged small section of the meninges included to illustrate a physiological coupling of trigemino-vascular and postganglionic nerve endings at this level. Kari C Toverud owns the copyright to the image. Printed with permission from © Kari C. Toverud.

In the current view on migraine pathophysiology, the transmission of nociceptive information through trigeminal fibres from meninges and extra- and intracranial vessels to the spinal trigeminal nucleus plays a crucial role in the pain phase of the migraine attack. ^1, 6^ This pathway, known as the trigemino-vascular system, constitutes the afferent part of the trigeminal-autonomic brainstem reflex ( Figure 1), a complex reflex arc that couples trigemino-vascular activation with an autonomic response underlying, amongst other, the cranial autonomic features observed in several primary headache disorders. ^7, 8^ The efferent part of this reflex pathway consists of parasympathetic fibres from the superior salivatory nucleus that synapse extracranially, in the sphenopalatine ganglion (SPG). Not only does the activation of the SPG trigger autonomic symptoms, but the postganglionic fibres from the SPG also exert effects on meningeal and intracranial blood vessels, thereby establishing a physiological connection with the trigeminal-vascular system at this level. ^8^ Therefore, by pharmacologically modulating the SPG, it may be possible to interfere with the trigemino-autonomic brainstem reflex arc.

This clinical trial explores the therapeutic potential of a pharmacological blockade of the SPG, a hitherto sparsely explored preventive treatment approach in migraine. Synaptic transmission between pre- and postganglionic neurons is classical cholinergic utilizing hexamethonium-sensitive nicotine receptor. Acetyl choline (ACh) and SNAP25, a protein necessary for ACh release from vesicles in nerve endings, are present in human SPG. ^9^ Botulinum toxins block the presynaptic release of ACh by cleaving SNAP25. ^10^ Consequently, botulinum toxins have the potential to produce a long-lasting, reversible block of SPG neurotransmission, without affecting sensory or sympathetic neurotransmission as neither forms synapses in the SPG.

**Figure 2.**
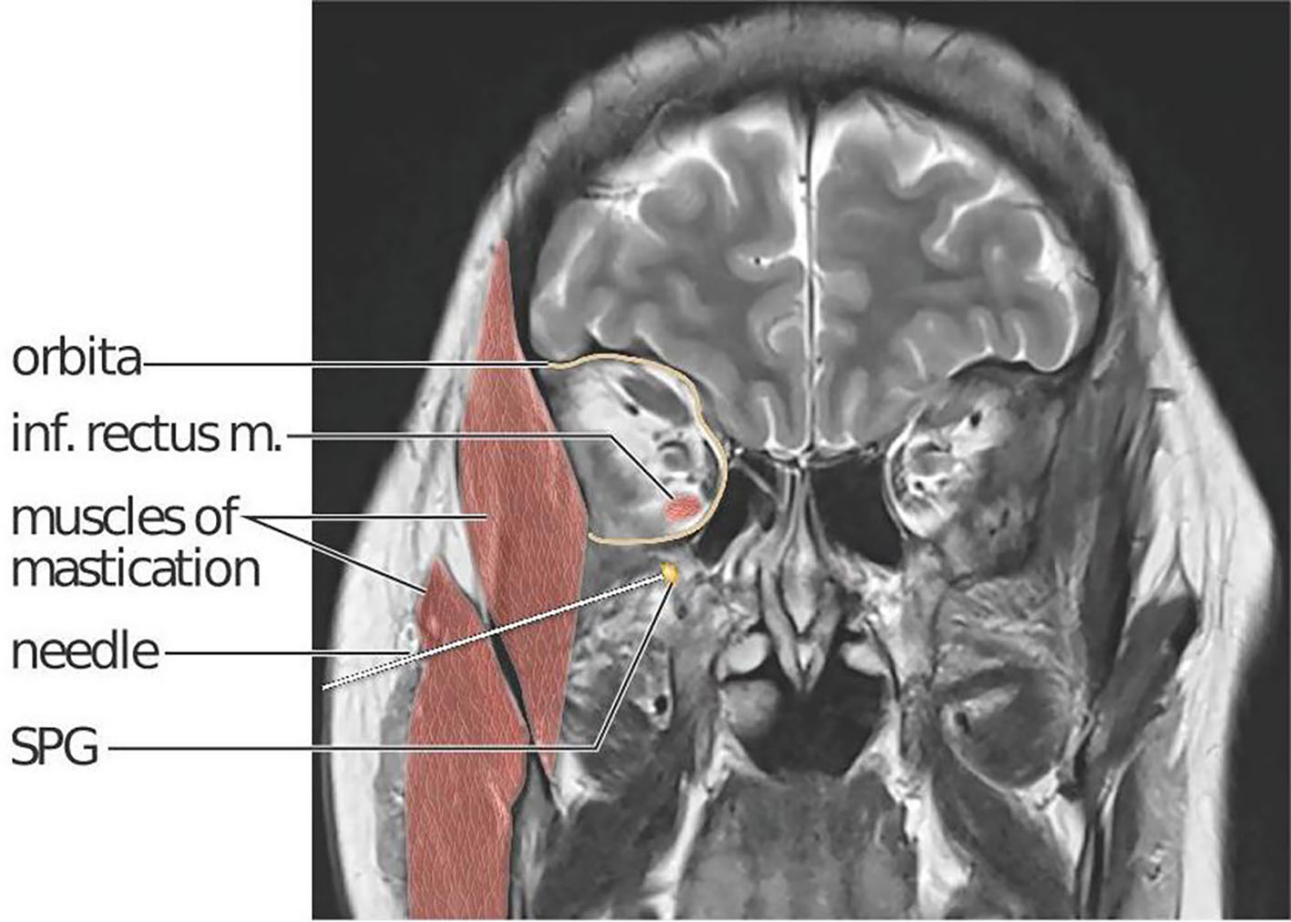
Coronal T2 MR scan section of the head including an illustration of the needle trajectory in a right lateral infrazygomatical injection approach. A needle penetrates skin and subcutaneous tissue, the masseter and temporal masticatory muscles (red) and ends at the right SPG (yellow). The SPG is located in the sphenopalatine fossa, just below the orbital (delineated in yellow). The sphenopalatine fossa and orbita communicate via the inferior orbital fissure. The inferior rectus muscle is highlighted to show the close anatomical relation between its orbital insertion and the location of the SPG. Kari C Toverud owns the copyright to the image. Printed with permission from © Kari C. Toverud.

In 1909, Sluder published the first report of treatment targeting the SPG, using intranasal administration. ^11^ More than a century later, only a limited number of studies exploring SPG-targeting interventions such as nasal lidocaine or bupivacaine and electrical or radiofrequency stimulation, exist. ^12^ Two randomised controlled studies on SPG stimulation in chronic cluster headache are published, demonstrating efficacy in aborting cluster headache attacks. ^13, 14^ The intervention requires surgery for device implantation and the device is currently not available. In chronic migraine, transnasal bupivacaine administration is the only SPG-targeting therapy studied in a blinded and placebo-controlled design. ^12, 15, 16^ The location of the SPG within the sphenopalatine fossa, situated deep within the facial structures behind the maxillary sinuses ( Figure 2), presents challenges for a non-invasive pharmacological treatment strategy targeting the SPG. The distance between the nasal mucosae and the SPG is a major obstacle for all methods applying non-invasive, intranasal administration. ^17^ The MiBlock trial addresses this challenge by employing a navigation-guided injection procedure to secure high precision targeting. The procedure can be performed in an office-based setting under local anaesthesia, utilizing a lateral (percutaneous) access rather than a transnasal approach. The procedure was piloted in an open-label, phase 2 study on treatment- resistant chronic migraine. Bilateral onabotulinumtoxinA injections in 10 participants were deemed safe and secondary migraine-related outcome measures were promising. ^18^ Indeed, eight out of 10 participants reported at least 50% reduction of moderate and severe headache days compared to baseline. Recently published real-world data on repeated treatments also provides favourable safety and efficacy data. ^19^

## Protocol

### Study objectives and endpoints

The primary objective of the MiBlock trial is to investigate the efficacy and safety of a single-session, bilateral navigation-guided onabotulinumtoxinA injection towards the SPG in participants with resistant chronic migraine.

### Primary efficacy endpoint

The primary efficacy endpoint variable is moderate and severe headache days, defined according to the guidelines of the International Headache Society. ^20^ Data capture is done prospectively as a patient-reported outcome in a study electronic headache Diary (eDiary). In the context of this study, a moderate or severe headache day is defined as a headache day with a recorded maximum pain intensity of ≥4 that lasts at least 4 hours or a day with a headache of any intensity which prompted the use of acute rescue medication. Rescue medications are defined as simple analgesics, triptans and opioids.

The primary efficacy endpoint is the change from the baseline in the number of moderate to severe headache days during weeks 5 to 8 following the intervention. The treatment effect will be assessed by comparing the average change in the treatment group to that in the placebo group. Post-injection weeks 5-8 are selected based on pilot study efficacy data suggesting that the frequency reduction the trial is designed to evaluate, reaches a plateau in this four-week period. ^18, 19, 21^

### Secondary efficacy endpoints

The statistical analysis plan (SAP) highlights three key secondary endpoint variables; number of migraine days, headache intensity and number of 30% responders. Other secondary endpoint variables in this study are cumulative hours of moderate and severe pain intensity, days with rescue medication and quality of life measures. Several prespecified exploratory endpoints will also be analysed, including number of crystal-clear headache-free days, number of 50% responders, 75% responders and 100% responders, change in autonomic symptoms and patient-reported outcomes questionnaires.

### Primary safety endpoint

Definition for safety reporting in this trial, including the definition of adverse event (AE) and serious adverse even (SAE), adheres to the European medicines agency standard. ^22^ Only treatment-emergent adverse events (TE-AE) and SAEs (TE-SAE) are collected. The primary safety endpoint is the occurrence of TE-AE and TE-SAE which will be compared between the placebo and the treatment arm.

## Methods

### Study design

This study is a multi-centre, randomised, quadruple-blinded, placebo-controlled trial. The study is investigator-initiated, exclusively publicly funded, and sponsored by a public hospital. It is designed according to the guidelines of the International Headache Society. ^20^ The trial is conducted entirely within the framework of the public healthcare system in Norway, which also reimburses travel and accommodation expenses. In case a co-payment is required, the study will reimburse such expenses. No payment is made to the study subjects for their participation.

### Study outline and recruitment

**Figure 3.**
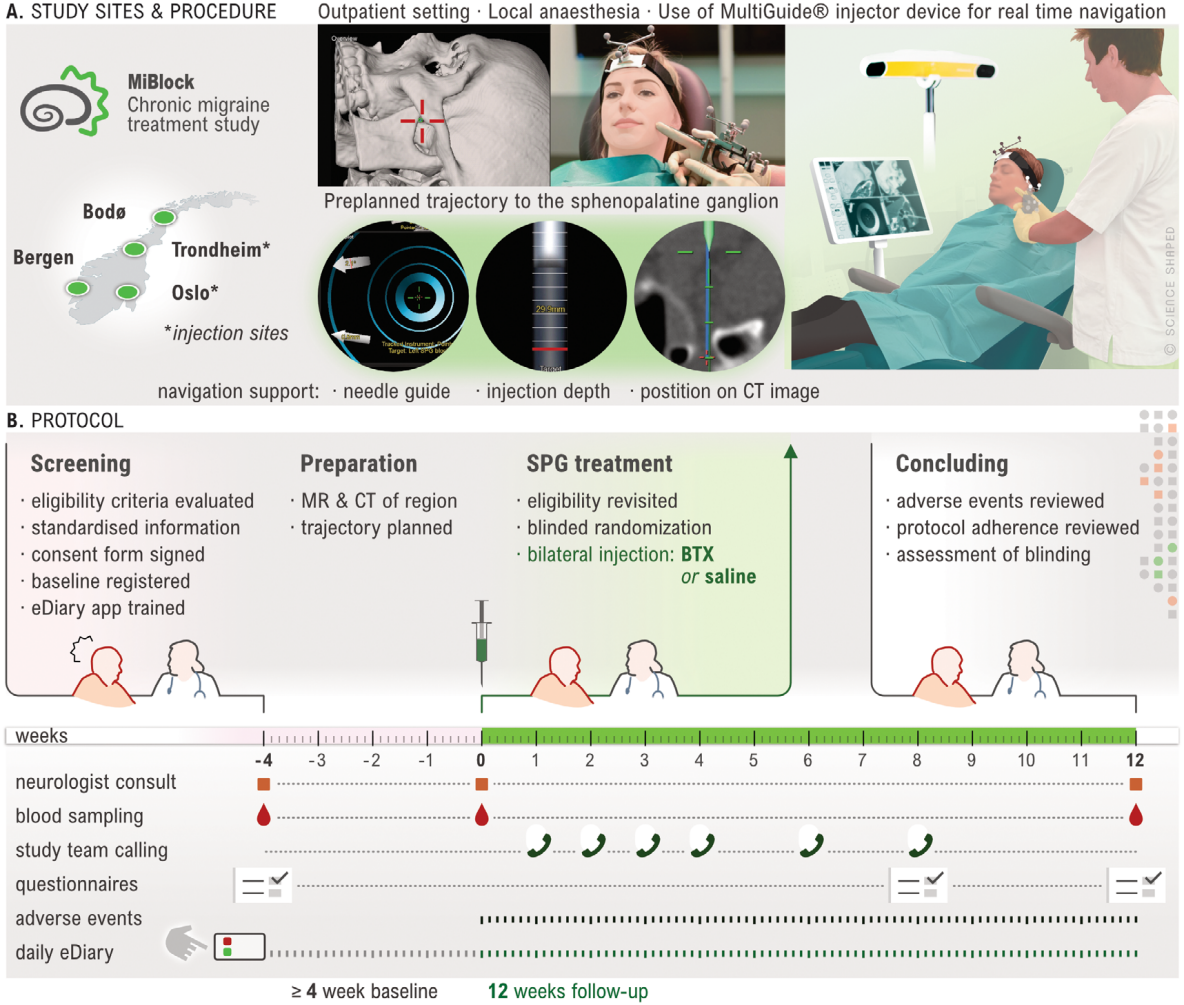
Illustration of study procedures. **A.** The study includes participants at 4 sites, but injections sites are Trondheim and Oslo only. Injections are done in an office-based setting. A preplanned injection trajectory with a percutaneous access and real time navigation guides the injection. **B.** Illustration of study progression for each participant. A screening visit followed by a 28-day minimum baseline period during which MR and CT scans are acquired. Follow rescreening, a single-session bilateral injection is performed, and a 12-week follow up initiated. Only AE occurring during injection or the follow up period (i.e. treatment emergent) are registered. Created by Ellen Tenstad. Printed with permission from © Ellen Tenstad @ScienceShaped.

An overview of the study procedures is provided in Figure 3. Participants are recruited from four general neurology clinics located at university hospitals in Oslo, Trondheim, Bergen, and Bodø. Each site represents one of the four administrative healthcare regions in Norway, enabling nationwide participation. Participants may be recruited directly at the site, referred from other neurology departments across Norway, referred by their general practitioner, or may initiate contact themselves with the recruitment sites. Study information is made available at institution and governmental webpages and social media platforms during the recruitment period.

Study participation is commenced by an inclusion visit followed by a baseline period of at least 28 days. Participants are instructed to keep a daily electronic headache diary (eDiary) for the entire duration of the study. At visit 2, prior to randomisation, a formal re-evaluation of study eligibility including a review of eDiary adherence and headache frequency, is performed. Participants assessed ineligible up to or at this point, are considered screening failures and excluded from the study. Treatment allocation follows a 1:1 ratio, with a computer-generated randomisation scheme applying block randomisation with randomly selected block sizes. CT and MR scans of the region of interest are performed prior to the injection procedure to allow trajectory planning and a navigation-guided injection. The follow-up period of 12 weeks ends with a final visit, which concludes study participation. At the end of this visit, participants and investigator are asked to assess blinding.

The inclusion visit is conducted in accordance with a dedicated standard operating procedure (SOP), which standardizes both the sequence and the content of evaluations performed by the investigator. Patients receive a written copy of the study’s informed consent form (ICF) prior to the visit. The session begins with a review of eligibility criteria. For patients who meet these criteria, the investigator then delivers scripted, standardized study information orally, supported by 10 illustrated cards. This approach ensures that all participants receive the same essential information regarding the study design, procedures, and safety profile. Patients are encouraged to ask questions during and after this part of the session. Only at this stage, if the investigator deems the patient eligible and adequately informed, are patients invited to sign the ICF, formally consenting to participate in the study. The investigator also signs the ICF to confirm that the information was provided in accordance with the SOP governing this visit.

### Eligibility criteria

Inclusion and exclusion criteria are listed in Table 1 ^32^ (Extended data). Eligible patients are adults 18-75 years of age, with resistant chronic migraine at time of inclusion; chronic migraine for 1 year prior to inclusion and debut of episodic migraine before the age of 50, and chronic migraine before the age of 65. Chronic migraine is defined according to ICHD-3-criteria as more than 3 months with 15 or more headache days each month, of which at least 8 days per month have pain and associate symptoms that comply with the migraine criteria. ^23^ The definition of resistant adheres to that of the European headache federation consensus statement of 2020 ^24^ and is in the study protocol operationalized as insufficient treatment effect, contraindication(s) or intolerable side effect(s) of at least three preventive medications from at least two prespecified drug classes.

Study treatment can be an add-on to a stable prophylactic regimen, provided it is kept unaltered during the entire trial participation and a minimum of 3 months before inclusion. The only exception is that use of botulinum toxin for any indication (including cosmetic) is not allowed for a period of four months prior to inclusion and during the study. Concomitant use of monoclonal antibody therapies targeting the CGRP system is allowed, but require a stable dose corresponding to 5 half-lives, i.e. a minimum of 20 weeks, prior to inclusion. Erenumab, fremanezumab, galcanezumab and eptinezumab are available in Norway. To date, atogepant is the only gepant reimbursed in Norway as a preventive treatment, but use of all gepants as prophylactic (in stable dose) or abortive drug is allowed. No restrictions on abortive medication use exist except for limiting opioids to 3 days per month. Participants cannot have secondary headaches, except for non-opioid medication overuse headache. Participants on antithrombotic medication could be considered for study participation if the treatment can be paused. In the case of monotherapy with acetylsalicylic acid, a pause is not required. A medical history with non-response to more than 6 preventive drugs from 7 predefined drug classes prior to participation is a cause for exclusion. As previous headache diary data may be unavailable, this assessment is at the investigators discretion. A patient-reported moderate response or intolerable side effect to a drug, is not classified as non-response to the drug.

### Woman of childbearing potential

The trial complies with the “Recommendations related to contraception and pregnancy testing in clinical trials” by the Clinical Trials Facilitation and Coordination group of Heads of Medicines Agencies (HMA). ^25^ Woman of childbearing potential (WOCBP) are required to take pregnancy tests at visit 1 and 2 and use highly effective contraception (HEC) as defined in the in recommendation the first 4 weeks post-injection. The Competent authority authorized one exception to the recommendation document, in that sexual abstinence from heterosexual intercourse during the first 4-week period post-injection could be regarded as HEC, albeit not the preferred and usual lifestyle of the subject.

### Study intervention

**Injection procedure**

The interventional procedure in MiBlock is a single session, bilateral, percutaneous injection towards the SPG performed using the MultiGuide® device aided by image-guided navigation. The MultiGuide® is a novel medical device developed by the research group from St. Olav’s Hospital and NTNU and holds a CE-label for the procedure. A commercially available surgical navigation system is used to plan an injection trajectory and provide real-time guidance of needle placement. The needle entry can either be supra- or infrazygomatical, depending on which trajectory provides best access to the sphenopalatine fossa. An example of an infrazygomatical trajectory is illustrated in Figure 2.

The intervention is performed at visit 2 in an outpatient setting at St. Olav’s University Hospital (Trondheim) and Oslo University Hospital by dedicated consultant neurosurgeons trained according to the protocol. Under local anaesthesia a small incision, approximately 2 mm wide, is made in the skin prior to needle insertion. The participant is observed for at least one hour following the procedure.

**Blinding and investigative medicinal product**

This study employs rigorous allocation concealment and blinding strategies referred to as quadruple-blinding. This involves blinding of the (1) participants and (2) investigators (double blinding), in addition to blinding of the (3) statistician/outcome assessor, and (4) the pre-specified order in which the statistical analysis will be performed including preplanned tables for the analysis subject to blinded analysis.

All personnel involved in the injection procedure were blinded to participant allocation. Designated unblinded study staff (“Unblinded Study Drug Coordinators”, USDCs) were automatically informed of each participant’s randomisation status via personal secure email notification. Based on this, the USDC prepared two sterile syringes (2 mL Omnifix Luer Lock, B. Braun, Germany) containing either:

1. onabotulinumtoxinA 25 units (Botox®, Allergan/AbbVie, USA), diluted in 0.5 mL sterile 0.9% sodium chloride injection solution (B. Braun, Germany), or
2. 0.5 mL sterile 0.9% sodium chloride solution (B. Braun, Germany) only.

All substances were sourced from the outpatient clinic’s medication stock; no catalogue numbers were available. Syringes were filled under sterile conditions following a standardized SOP, labeled with participant ID and initials, and sealed in sterile packaging. The USDC had no contact with participants or study staff involved in injections and placed the syringes in a predefined, access-controlled storage area for blinded staff to collect. Syringes were used within six hours of preparation, as per stability guidance from the manufacturer and SOP. The USDC was responsible for maintaining and storing the Drug Accountability Log, in which the participant ID number and initials, batch number, expiry date, and the date and time of preparation were recorded.

To assess the integrity of the double-blind design at the final visit, each participant and investigator is asked to indicate the treatment allocation of the subject, choosing from one of three options: Verum, Placebo, or “Don’t know”.

According to the SAP, independent statisticians will perform a blinded analysis without knowledge of the actual treatment allocation, using a dataset in which randomisation status is coded as Group A and Group B. The blinded analyses will follow a pre-specified order to minimize the risk of unintentional unblinding before completion. The result table templates for the blinded analysis are pre-specified for the statistical analysis report, and included as an appendix to the SAP, that will be finalized prior to the study data lock.

### Procedures for unblinding

The Department of Neurology at the University Hospital in Trondheim provides a 24/7 emergency telephone service for all study participants. The on-call neurologist is responsible for responding to calls and may initiate unblinding if necessary. To enable continuous unblinding availability, a research nurse at the Department of Neurology in Trondheim, who is otherwise not involved in the study and has no contact with study participants, is automatically notified by email at the time of randomisation. This nurse prints the randomisation outcome for each participant and places it in a sealed envelope labelled only with the participant’s unique identification number. These envelopes are stored in a secure archive at the Department of Neurology, accessible to the on-call neurologist at all times.

Given that the effects of onabotulinumtoxinA cannot be reversed and each participant receives a single bilateral injection, the SOP states that any unblinding should, if feasible, be discussed in advance with the Coordinating Investigator.

### Data collection

A source data list specifies what constitutes source data. Data on visits, eligibility assessments, current medical history, and medication use, including headache diagnoses, and previous use of preventive headache medication, in addition to safety data has its source documentation in the patient electronic medical records (EPJ). Data handling is done according to the General Data Protection Regulation and institution guidelines. Data will be stored for 25 years after study completion for control purposes as required by Norwegian law.

*eCRF*

An electronic Case Report Forms (eCRF) applying the WebCRF3 software, was developed for this study. The webCRF3 software is provided and maintained by the research department at St Olav’s university hospital. Data collected at study visits, telephone consultations and safety information are entered. Data is stored in a pseudo-anonymous manner, by which each study participant is recognizable by his/her unique participant study identification (ID) numbers.

*eDiary*

Evaluation of the primary endpoint is solely based on patient-reported daily eDiary entries collected prospectively during the entire trial via a mobile phone application (app). The participant must make entries every day, including days when they do not experience headache. The tool is designed such that when participants report having headache, they will be prompted to answer additional questions about the maximum pain intensity, duration of headache, duration of headache of intensity ≥4, associated symptoms including aura, abortive medication use and self-reported identification of headache as migraine and/or other primary headaches. On days without headache, questions related to aura and potential prodromal or postdromal symptoms are asked. Regardless of headache status, data on impairment at work or in other activities, and potential adverse events are collected daily.

Investigators can enter an online dashboard that displays if daily entries to the diary have been done or not. The dashboard allows identification of participants not adhering to the diary. The system also prepares a report of patient-reported potential adverse events, to be followed up during the study. The eDiary app allows registration in a three-day interval (present day, and the two previous days). A back-up paper diary is provided to participants in case of technical problems.

*Safety data*

Safety data are collected prospectively through the eDiary app and through interviews as part of study visits and phone consultations. Study personnel register all AEs in a designated section of the eCRF.

Periprocedural adverse events is defined as AEs starting during or within one-hour post-injection, and are assessed for causal relationship to IMP, medical device and local anaesthesia. All other adverse event occurring in the 12 weeks postinjection are assessed for causal relationship to IMP and the medical device only. All adverse events will be coded using the MedDRA version 28.0 and severity is graded according to the Common Terminology Criteria for Adverse Events (CTCAE) version 5.0. Causality assessments and severity grading are performed by investigators at each site. Coding is performed according to the project management plan by one blinded medical monitor.

Previously reported adverse events to SPG-injections ( Table 2) and Summary of Product Characteristics (SmPC) for onabotulinumtoxinA are considered expected adverse events.

**Table 2.** Expected adverse events (AE) based on data available at the time of study initiation. Number of patients (n) = 93 (patients with adverse events (AEs), n = 59).

Adverse event	Number of patients
Pain, swelling and numbness (face, cheek, temporomandibular joint, incision site, teeth, nose, palatine)	29
Jaw problems (reduced opening, muscle weakness, chewing problems)	23
Headache	3
Visual disturbances (blurring, diplopia, accommodation problems)	17
Dry eye	2
Facial asymmetry	7
Bleeding (incision site)	1
Nasal bleeding	2
Facial asymmetry, hospitalization for imaging (SAE) ^a^	1
Tearing	2
Mild dysphagia	1
Nasal obstruction	1
Rhinorrhea	1
Hematoma (incision site)	1
Tinnitus	1
Nausea	1
**Total AE (SAE)**	**93 (1)**

^a^ Serious adverse event = SAE.

*Acceptability of treatment*

Short-lasting periprocedural pain and procedure duration are documented in the eCRF. Immediately after the needle is withdrawn following the second injection, the participant is asked to rate their pain using an 11-point Numerical Rating Scale (NRS) in the following way: (1) Rate the maximum pain experienced during the injection; (2) Rate the current level of pain. Additionally, at the final visit, the participant is asked whether they would be willing to undergo the injection again. Treatment acceptability will be evaluated based on this data.

*Device related events*

Any device deficiencies are collected after the injection procedure. Any adverse event that is deemed to have a possible, probable or causal relationship to the medical device is also registered as an adverse device event.

*Patient-reported outcomes questionnaires*

The following questionnaires are used in the study. All copyrighted instruments were used with proper permission or license. References to copyright and licensing are provided for each tool below:

• Headache Impact Test™ (HIT-6™; © 2001 QualityMetric Incorporated and the GlaxoSmithKline Group of Companies. All rights reserved). ^26^ The HIT-6™ was licensed (Lic.no. QM49738) under a non-commercial license agreement for this study. All rights reserved.
• Migraine-Specific Quality-of-Life Questionnaire Version 2.1© (MSQv2.1©). ^27^ The MSQ is copyrighted and registered with the United States Library of Congress by Glaxo Wellcome, Inc. Permission for use obtained via Mapi Research trust.
• Work productivity and activity impairment: Migraine v2.1 (WPAI:M v2.1). ^28^ Instrument not copyrighted according to the author, Margaret C. Reilly. Provided via Mapi Research trust.
• Hospital Depression and Anxiety Scale© (HADS©), ^29^ copyright © R.P. Snaith and A.S. Zigmond, 1983, 1992, 1994. Permission for use obtained via Mapi Research trust.
• Patients’ global impression of improvement questionnaire ^30^ are public domain. PGI-C has undergone a formal translation process to Norwegian according to linguistic validation methodology.

Patients’ global impression of improvement (PGI-I) and of change (PGI-C) are registered at week 1, 2, 3, 4, 6, 8 and 12 postinjection. Depression and anxiety experienced by participant are evaluated with the 14-item Hospital Depression and Anxiety Scale© (HADS©) at visit 1 and post-intervention week 8 and week 12. At the same milestones, migraine-related quality of life measures and headache disability are mapped with the 6-question Headache impact test™ (HIT-6™) and the 14-question Migraine-Specific Quality-of-Life Questionnaire© Version 2.1 (MSQv2.1©). Work productivity and activity impairment: Migraine v2.1 (WPAI:M v2.1) questionnaire will be completed at visit 1 and weeks 12 post-intervention.

*Blood samples and biobanking*

Blood samples for the analysis of haemoglobin, leukocytes with differential count, thrombocytes, and C-reactive protein (CRP) are collected at visits 1, 2, and 3 ( Figure 3). At these same visits, participants may also provide additional consent to have blood drawn for biobanking. The latter follows a dedicated SOP and is stored at a research biobank at Oslo University Hospital.

*Data management plan*

Data management is guided by the data management plan. Data verification of questionnaire and haematology report data in the eCRF will be conducted on 10% of participants at each site.

### Statistical analysis

**Research hypothesis**

This trial is designed to investigate the superiority of onabotulinumtoxinA compared to placebo when injected into the SPG using the MultiGuide® device with respect to the number of moderate to severe headache days:

• The null hypothesis is that the average change from baseline in the number of moderate to severe headache days during weeks 5-8 post-injection does not differ between participants receiving onabotulinumtoxinA or placebo.
• The alternative hypothesis is that the average change from baseline in the number of moderate to severe headache days during weeks 5-8 post-injection differs between participants receiving onabotulinumtoxinA or placebo.

**Sample size**

The MiBlock trial has a confirmatory statistical strategy that pre-specifies just one single hypothesis related to the primary endpoint. To that end, the sample size was calculated by a professional, independent biostatistician based on the data from the pilot study. ^18^ The standard deviation (SD) for the change in number of headache days per week between baseline and week 5 – 8 was 7. To detect an efficacy size of 3.5 less moderate to severe headache days between active and placebo groups in weeks 5 – 8, provided 80% power to reject the null hypothesis at 5% level nominal of significance and assuming a drop-out rate of 25%, a total of 170 patients will be randomised to treatment in this study.

**Subject adherence, protocol deviations and violations**

The definition of protocol deviation and violation is based on the following statement ^31^ and should in this study cover all incidences of participant non-adherence to study procedures after enrolment (ie. post-randomisation). Protocol violations in this study are headache diary nonadherence defined as <90% adherence and/or alterations in headache preventive treatment during trial participation. Electronic headache diary adherence will be calculated at 4-week (28 days) intervals. Protocol violators are excluded from the per protocol analysis.

### Statistical analysis

The main analysis is planned when all participants have concluded the 12-week follow-up after the injection and the database has been locked. All statistical analyses are pre-planned and will be described in a SAP finalised prior to data lock. The SAP will also include a preplanned order for data analysis, as well as the results table templates for the blinded analysis. The order of data analysis is defined a priori in order to ensure that the main efficacy analyses are completed without the risk of unblinding which may occur when analysing the safety data.

The full analysis set (FAS) will include all randomised participants who received the injection. To adhere to the intention-to-treat ideal for the FAS population, participants that undergo unsuccessful injections (i.e. Investigative medicinal product (IMP) not administered bilaterally or unilaterally) are included in the FAS.

The FAS will be used for all efficacy analyses. Briefly, for the primary outcome, a mixed logistic regression model will be used to estimate the difference in the change from baseline in mean monthly headache days between the treatment and placebo group. The model will include the presence or absence of headache on each day with recorded information from baseline until 12 weeks after the injection as a dependent binary variable, treatment allocation, time-period (baseline, week 1-4, week 5-8 and week 9-12) and an interaction term between treatment allocation and post-injections time-periods are included as categorical fixed effects, and with participant ID as a random effect. Site of inclusion will also be assessed as a potential random effect prior to unblinding of the statistician to treatment allocation. All available data will be included in this analysis, regardless of proportion of eDiary days completed. The analysis of secondary outcomes will use the same strategy, although using mixed linear regression models for continuous outcomes. Additionally, sensitivity analyses for the primary and secondary outcomes will be performed, which will include restriction analysis to the subgroup of participants with technically successful bilateral injections and the per-protocol set.

A prespecified assessment of the double blinding is to be included in the SAP and will be included in the statistical analysis report.

### Study monitoring

The study is monitored by independent monitors according to a monitoring plan developed based on their own project risk analysis. In addition, two independent, unblinded monitors are assigned to the two injection sites to monitor the drug accountability log.

An independent data safety monitoring committee consisting of one consultant anaesthesiologist with previous service on ethical boards, one neurologist with headache sub-specialisation and one biostatistician will assess safety at 10%, 25%, 50% and 75% of treated participants and advise the sponsor on the safety profile.

### Data access

Upon data lock following study completion, only the blinded study statistician will have access to the complete dataset, in accordance with procedures prespecified in the SAP. Once the analyses outlined in the SAP have been completed, the full dataset will be made available to all study investigators to facilitate preparation of the results for publication and to allow their independent review of the data. No contractual or other restrictions apply to this process, beyond compliance with applicable data protection regulations.

### Dissemination

The results of this trial will be published in international open access peer-reviewed medical journals and presented at relevant scientific meetings. The SAP and statistical analysis report will be made available upon publication. Publication will adhere to the Vancouver guidelines.

## Discussion

To the best of our knowledge, MiBlock is the first study to evaluate the efficacy of a bilateral onabotulinumtoxinA blockade towards the SPG in a treatment-resistant chronic migraine population. The eligible study participants represent a heavily burdened patient population in need of novel treatment approaches. Despite recent progress in treatment options, including monoclonal antibodies and gepants targeting the CGRP system, a significant proportion is still inadequately relieved of their migraine. For them, blockage of synaptic transmission in the SPG and thus modulation of the trigemino-autonomic reflex constitutes a novel treatment approach. Pilot studies in both cluster headache and chronic migraine show an acceptable safety profile and promising efficacy data. ^18, 21^ Recently published real-world data from an open-label treatment setting suggest that the therapeutic effect may be sustained over repeated injections, further supporting the rationale for evaluating this approach in a placebo-controlled trial. ^19^

This study is designed to allow a robust evaluation of efficacy. To address the presumed important placebo-response associated with interventional procedures, a placebo arm is required in addition to a resilient quadrupled blinding scheme. A residual risk of unblinding due to the occurrence of adverse events (interpreted as possible onabotulinumtoxinA effects), such as transient facial weakness or diplopia, unfortunately, cannot be avoided. To explore their effect on the blinding regimen, data to assess the blinding is collected at the final study visit.

Additional strengths of the study include precise targeting of the SPG, applying commercially available navigation systems in clinical use, and designated consultant neurosurgeons performing the procedure at two sites.

The study design poses ethical challenges as participants are required to keep their preventive medication stable during their trial participation, with a 50% chance of receiving placebo. In the design of the study care has been given to provide sufficient and standardised information to eligible candidates, underscoring these issues and allowing sufficient time to reflect on their decision to participate.

## Study status

The date of first enrolment was 25.10.2019, but with significant delays in recruitment from March 2020 due to the COVID-19 pandemic, including the need to temporarily close the inclusion. Recruitment ended February 2025.

## Ethical consideration

The disease burden of this pain condition renders this group vulnerable, underscoring the importance of properly conducted trials. Several measures are taken to ensure that patients are sufficiently informed before consenting to trial participation. Patients receive written information that includes the possibility of not having an effect of the IMP (or receiving placebo), and the possible complications of the intervention, prior to the first visit. A scripted review of study information with illustration is provided by investigators to all participants at visit 1, prior to providing informed consent. Participants were informed that the study protocol does not include an extension phase and that no opportunity for repeated treatment is provided within the framework of this study. All participants gave written informed consent before any study procedures were initiated.

Participants are insured through the Norwegian pharmaceutical liability Insurance (Legemiddelansvarsforsikringen) and the Norwegian System of Patient Injury Compensation (Norsk pasientskadeerstatning).

The trial adheres to the Declaration of Helsinki. The study protocol and the informed consent forms was reviewed and approved by the Norwegian ethics committee (ref 2018/2161, date of approval 8 ^th^ of February 2019) and the Norwegian medical agency (ref nr. 18/16600-11, date of approval 20 ^th^ of august 2019) prior to study initiation. Substantial amendments to the protocol are subject to review prior to implementation.

Wergeland, Tore (2025). Table 1, Eligibility criteria for participation in the MiBlock trial. figshare. Journal contribution. https://doi.org/10.6084/m9.figshare.28930940.v1

## Data Availability

No data is associated with this article. 1. Figshare: Table 1, Eligibility criteria for participation in the MiBlock trial, https://doi.org/10.6084/m9.figshare.28930940.v1 ^32^
This project contains the following underlying data:
Table 1 Data is available under CC BY 4.0 license 2. Figshare: SPIRIT MiBlock.pdf, https://doi.org/10.6084/m9.figshare.28806617.v1 ^33^
This project contains the following underlying data: SPIRIT checklist Data is available under CC BY 4.0 license 3. Figshare: Informed consent form - the Miblock trial, https://doi.org/10.6084/m9.figshare.28806665.v1 ^34^
This project contains the following underlying data: Consent form Data is available under CC BY 4.0 license 4. Figshare: Informed consent form (english translation) - The MiBlock trial, https://doi.org/10.6084/m9.figshare.29165627.v1 ^35^
This project contains the following underlying data: Translation of consent form Data is available under CC BY 4.0 license

## References

[1] Goadsby PJ Holland PR Martins-Oliveira M : Pathophysiology of Migraine: A Disorder of Sensory Processing. *Physiol. Rev.* 2017;97:553–622. 10.1152/physrev.00034.2015 28179394 PMC5539409

[2] Charles A : Migraine. *N. Engl. J. Med.* 2017;377:553–561. 10.1056/NEJMcp1605502 28792865

[3] Steiner TJ Stovner LJ Vos T : Migraine is first cause of disability in under 50s: Will health politicians now take notice? *J. Headache Pain.* 2018;19:17. 10.1186/s10194-018-0846-2 29468450 PMC5821623

[4] Natoli JL Manack A Dean B : Global prevalence of chronic migraine: A systematic review. *Cephalalgia.* 2010;30:599–609. 10.1111/j.1468-2982.2009.01941.x 19614702

[5] Tepper SJ : History and Review of anti-Calcitonin Gene-Related Peptide (CGRP) Therapies: From Translational Research to Treatment. *Headache.* 2018;58 Suppl 3:238–275. 10.1111/head.13379 30242830

[6] Dodick DW : A Phase-by-Phase Review of Migraine Pathophysiology. *Headache.* 2018;58 Suppl 1:4–16. 10.1111/head.13300 29697154

[7] May A Goadsby PJ : The trigeminovascular system in humans: Pathophysiologic implications for primary headache syndromes of the neural influences on the cerebral circulation. *J. Cerebr. Blood F. Met.* 1999;19:115–127. 10.1097/00004647-199902000-00001 10027765

[8] Moller M May A : The unique role of the trigeminal autonomic reflex and its modulation in primary headache disorders. *Curr. Opin. Neurol.* 2019;32:438–442. 10.1097/WCO.0000000000000691 30865010

[9] Steinberg A Frederiksen SD Blixt FW : Expression of messenger molecules and receptors in rat and human sphenopalatine ganglion indicating therapeutic targets. *J. Headache Pain.* 2016;17:78. 10.1186/s10194-016-0664-3 27587062 PMC5009057

[10] Wheeler A Smith HS : Botulinum toxins: mechanisms of action, antinociception and clinical applications. *Toxicology.* 2013;306:124–146. 10.1016/j.tox.2013.02.006 23435179

[11] Sluder G : The anatomical and clinical relations of the sphenopalatine (Meckel’s) ganglion to the nose and its accessory sinuses1909. 1909.

[12] Ho KWDP Przkora R Kumar S : Sphenopalatine ganglion: block, radiofrequency ablation and neurostimulation - a systematic review. *J. Headache Pain.* 2017;18:118. 10.1186/s10194-017-0826-y 29285576 PMC5745368

[13] Schoenen J Jensen RH Lanteri-Minet M : Stimulation of the sphenopalatine ganglion (SPG) for cluster headache treatment. Pathway CH-1: A randomized, sham-controlled study. *Cephalalgia.* 2013;33:816–830. 10.1177/0333102412473667 23314784 PMC3724276

[14] Goadsby PJ Sahai-Srivastava S Kezirian EJ : Safety and efficacy of sphenopalatine ganglion stimulation for chronic cluster headache: A double-blind, randomised controlled trial. *Lancet Neurol.* 2019;18:1081–1090. 10.1016/S1474-4422(19)30322-9 31701891

[15] Cady R Saper J Dexter K : A double-blind, placebo-controlled study of repetitive transnasal sphenopalatine ganglion blockade with tx360((R)) as acute treatment for chronic migraine. *Headache.* 2015;55:101–116. 10.1111/head.12458 25338927 PMC4320756

[16] Cady RK Saper J Dexter K : Long-term efficacy of a double-blind, placebo-controlled, randomized study for repetitive sphenopalatine blockade with bupivacaine vs. saline with the Tx360 device for treatment of chronic migraine. *Headache.* 2015;55:529–542. 10.1111/head.12546 25828648 PMC6681144

[17] Crespi J Bratbak D Dodick D : Measurement and implications of the distance between the sphenopalatine ganglion and nasal mucosa: A neuroimaging study. *J. Headache Pain.* 2018;19:14. 10.1186/s10194-018-0843-5 29442191 PMC5811417

[18] Bratbak DF Nordgard S Stovner LJ : Pilot study of sphenopalatine injection of onabotulinumtoxinA for the treatment of intractable chronic migraine. *Cephalalgia.* 2017;37:356–364. 10.1177/0333102416648328 27154997 PMC5394468

[19] Simmonds L Jamtoy KA Aschehoug I : Open label experience of repeated OnabotulinumtoxinA injections towards the sphenopalatine ganglion in patients with chronic cluster headache and chronic migraine. *Cephalalgia.* 2024;44:3331024241273967. 10.1177/03331024241273967 39165124

[20] Tassorelli C Diener HC Dodick DW : Guidelines of the International Headache Society for controlled trials of preventive treatment of chronic migraine in adults. *Cephalalgia.* 2018;38:815–832. 10.1177/0333102418758283 29504482

[21] Bratbak DF Nordgard S Stovner LJ : Pilot study of sphenopalatine injection of onabotulinumtoxinA for the treatment of intractable chronic cluster headache. *Cephalalgia.* 2016;36:503–509. 10.1177/0333102415597891 26232105 PMC4853809

[22] agency Em: NOTE FOR GUIDANCE ON CLINICAL SAFETY DATA MANAGEMENT: DEFINITIONS AND STANDARDS FOR EXPEDITED REPORTING (CPMP/ICH/377/95). 1995.

[23] Olesen J : International Classification of Headache Disorders. *Lancet Neurol.* 2018;17:396–397. 10.1016/S1474-4422(18)30085-1 29550365

[24] Sacco S Braschinsky M Ducros A : European headache federation consensus on the definition of resistant and refractory migraine: Developed with the endorsement of the European Migraine & Headache Alliance (EMHA). *J. Headache Pain.* 2020;21:76. 10.1186/s10194-020-01130-5 32546227 PMC7296705

[25] Group CCTC: Recommendations related to contraception and pregnancy testing in clinical trials Version 1.2. 2024. Reference Source

[26] Kosinski M Bayliss MS Bjorner JB : A six-item short-form survey for measuring headache impact: the HIT-6. *Qual. Life Res.* 2003;12:963–974. 10.1023/a:1026119331193 14651415

[27] Martin BC Pathak DS Sharfman MI : Validity and reliability of the migraine-specific quality of life questionnaire (MSQ Version 2.1). *Headache.* 2000;40:204–216. 10.1046/j.1526-4610.2000.00030.x 10759923

[28] Reilly MC Zbrozek AS Dukes EM : The validity and reproducibility of a work productivity and activity impairment instrument. *PharmacoEconomics.* 1993;4:353–365. 10.2165/00019053-199304050-00006 10146874

[29] Zigmond AS Snaith RP : The hospital anxiety and depression scale. *Acta Psychiatr. Scand.* 1983;67:361–370. 10.1111/j.1600-0447.1983.tb09716.x 6880820

[30] Guy W. National Institute of Mental Health (U.S.): Psychopharmacology Research Branch, Early Clinical Drug Evaluation Program : *ECDEU assessment manual for psychopharmacology.* Rockville, Md: U.S. Dept. of Health, Education, and Welfare, Public Health Service, Alcohol, Drug Abuse, and Mental Health Administration, National Institute of Mental Health, Psychopharmacology Research Branch, Division of Extramural Research Programs;1976. Rev.

[31] Bhatt A : Protocol deviation and violation. *Perspect. Clin. Res.* 2012;3:117. 10.4103/2229-3485.100663 23125964 PMC3487227

[32] Wergeland TW : Table 1, Eligibility criteria for participation in the MiBlock trial. *figshare. Journal contribution.* 2025. 10.6084/m9.figshare.28930940.v1

[33] Wergeland TW : SPIRIT MiBlock.pdf.Dataset. *figshare.* 2025. 10.6084/m9.figshare.28806617.v1

[34] Wergeland TW : Informed consent form - the Miblock trial. *figshare. Presentation.* 2025. 10.6084/m9.figshare.28806665.v1

[35] Wergeland TW : Informed consent form (english translation) - The MiBlock trial. *figshare. Journal contribution.* 2025. 10.6084/m9.figshare.29165627.v1

