# Supplementary material for: OnabotulinumtoxinA blockade of the sphenopalatine ganglion in treatment-resistant chronic migraine (MiBlock): rationale, design and study protocol for a multi-centre, investigator initiated, randomised, quadruple-blinded placebo-controlled trial
Source: F1000Res. 2026 Feb 5;14:564. Originally published 2025 Jun 6. [Version 2] doi: 10.12688/f1000research.164241.2 (PMC12921396; doi:10.12688/f1000research.164241.2)
Supplement: Peer review history

# Reviewer report: Reviewer response for version 2

**DOI:** 10.5256/f1000research.195621.r455690

**Published:** 2026-02-19

**Authors:** Keiko Ihara

**Affiliations:** 1 Department of Neurology, Mayo Clinic, Rochester, Minnesota, USA

I have reviewed the updated version and have no further concerns.

Is the study design appropriate for the research question?

Yes

Is the rationale for, and objectives of, the study clearly described?

Yes

Are sufficient details of the methods provided to allow replication by others?

Partly

Are the datasets clearly presented in a useable and accessible format?

Partly

Reviewer Expertise:

NA

I confirm that I have read this submission and believe that I have an appropriate level of expertise to confirm that it is of an acceptable scientific standard.

# Reviewer report: Reviewer response for version 2

**DOI:** 10.5256/f1000research.195621.r455691

**Published:** 2026-02-06

**Authors:** Lanfranco Pellesi

**Affiliations:** 1 University of Southern Denmark, Odense, Denmark

I am satisfied with the responses.

Is the study design appropriate for the research question?

Yes

Is the rationale for, and objectives of, the study clearly described?

Yes

Are sufficient details of the methods provided to allow replication by others?

Yes

Are the datasets clearly presented in a useable and accessible format?

Not applicable

Reviewer Expertise:

Clinical pharmacologist with an expertise in headache disorders

I confirm that I have read this submission and believe that I have an appropriate level of expertise to confirm that it is of an acceptable scientific standard.

# Reviewer report: Reviewer response for version 1

**DOI:** 10.5256/f1000research.180716.r443086

**Published:** 2026-01-07

**Authors:** Keiko Ihara

**Affiliations:** 1 Department of Neurology, Mayo Clinic, Rochester, Minnesota, USA

The authors provided a thorough study protocol on onabotulinumtoxinA blockade of the sphenopalatine ganglion in patients with chronic migraine. They specifically targeted those who have tried at least three preventive medications and do not have secondary headaches other than MOH. They chose a randomised, quadruple-blinded, placebo-controlled trial approach and specified randomisation, statistical analysis, and procedure protocol in detail. Several items that need to be specified are listed below.

 - Please clearly define 30%, 50%, and 75% responders. What metric would define these percentages?

 - How many times are patients allowed to enter eDiary data per day? How would they answer if they experienced multiple headaches in one day?

 - Would the patients be informed of migraine definition before starting their eDiary entry?

 - Would the patients be informed of potential AE types and their expected frequency? 

 - Regarding “stable” dosage of preventive medication during the trial, would the authors allow patients to switch between monthly and quarterly fremanezumab?

Is the study design appropriate for the research question?

Yes

Is the rationale for, and objectives of, the study clearly described?

Yes

Are sufficient details of the methods provided to allow replication by others?

Partly

Are the datasets clearly presented in a useable and accessible format?

Partly

Reviewer Expertise:

migraine, CGRP mAbs, real-world data

I confirm that I have read this submission and believe that I have an appropriate level of expertise to confirm that it is of an acceptable scientific standard, however I have significant reservations, as outlined above.

# Response

**Published:** 2026-01-22

**Authors:** Tore Wergeland

**Affiliations:** Department for neuromedicine, Norwegian University of Science and Technology Department of Neuromedicine and Movement Science, Trondheim, Trøndelag, Norway

*We thank the reviewer for highlighting the need for clarifications. We have addressed all concerns and updated the manuscript accordingly.*

**- Please clearly define 30%, 50%, and 75% responders. What metric would define these percentages?**

*Reply: In this trial, responder status is defined based on the percentage change in the number of moderate to severe headache days, comparing each 28-day post-intervention period with the 28-day baseline period.*

*Moderate to severe headache days are defined in accordance with International Headache Society (IHS) guidelines and as specified in the study protocol and statistical analysis plan (SAP). The ≥30% responder rate in week 5-8 is a prespecified secondary endpoint, while ≥50%, ≥75%, and 100% responder rates are prespecified exploratory endpoints.*

*To reduce the risk of uncertainty or misclassification of responder status due to incomplete data, responder endpoints will be calculated only for participants who have completed at least 90% of daily headache diary registrations in both 28-day periods used for the respective comparisons.*

**- How many times are patients allowed to enter eDiary data per day? How would they answer if they experienced multiple headaches in one day?**

*Reply: Participants are instructed to complete one eDiary entry per calendar day.*

*If a participant experiences multiple headache episodes within the same day, features such as intensity and duration are recorded collectively within the same daily entry. This approach is consistent with standard headache diary methodology and ensures that each calendar day is classified as either a headache day or a non-headache day for endpoint derivation. Participants was also asked to self-identify type of headache, and in case of several primary headaches occurring on one day, could identify this in the diary.*

**- Would the patients be informed of migraine definition before starting their eDiary entry?**

*Reply: First and foremost, as part of the eligibility assessment, the participants headache conditions was verified according to the ICHD3-criteria by a study investigator. As part of study onboarding, participants were trained in use of the eDiary, that explicitly askes participants to self-identify type of headache (migraine, TTH, cluster etc.) on days with headache.  In accordance with the eligibility criteria, participants who were unable to distinguish migraine from other headache types were excluded.*

**- Would the patients be informed of potential AE types and their expected frequency?**

*Reply: Yes. Participants were informed of both the types of potential adverse events (AEs) and their expected frequency during the informed consent process, in accordance with ethical and regulatory requirements. This information was provided through written material and standardized oral information.*

*Information on AE types and frequencies was based on reported findings from prior open-label pilot studies and accumulated clinical experience. Due to the open-label nature of these data, it is not possible with certainty to distinguish whether reported AEs are attributable to the injection procedure itself or to the investigational medicinal product. Thus, we could not relate AEs specifically to the IMP in the information provided.*

**- Regarding “stable” dosage of preventive medication during the trial, would the authors allow patients to switch between monthly and quarterly fremanezumab?**

*Reply: No. A switch between monthly and quarterly fremanezumab dosing during the trial would be considered a change in preventive treatment regimen and is therefore not permitted.*

*To be eligible for inclusion, participants must be on a stable preventive treatment regimen for at least the prespecified period prior to enrolment (5 months for monoclonal antibodies targeting the CGRP pathway), and this regimen must remain unchanged throughout trial participation.*

*Switching dosing intervals of fremanezumab would constitute a protocol violation. Such a change would result in designation as a screening failure if occurring prior to randomisation, and exclusion from the per-protocol analysis set if occurring post-randomisation.*

# Reviewer report: Reviewer response for version 1

**DOI:** 10.5256/f1000research.180716.r443087

**Published:** 2026-01-06

**Authors:** Lanfranco Pellesi

**Affiliations:** 1 University of Southern Denmark, Odense, Denmark

This protocol describes an investigator-initiated, multicentre, randomized, quadruple-blinded, placebo-controlled trial evaluating navigation-guided onabotulinumtoxinA injection into the sphenopalatine ganglion (SPG) for treatment-resistant chronic migraine. I have some comments:

 MAJOR COMMENTS

 - There is limited discussion of why SPG blockade should preferentially affect migraine frequency rather than cranial autonomic symptoms, which are more directly linked to SPG physiology. I suggest to expand the discussion to clarify the hypothesized mechanistic pathway linking SPG blockade to reduced migraine frequency, especially in patients already exposed to CGRP-targeting therapies.

 - The population is extremely heterogeneous, including patients on CGRP monoclonal antibodies, gepants (preventive and abortive), oral preventives and PREEMPT-style onabotulinumtoxinA (historical exposure). Patients with very high prior treatment failure (> 6 drugs) are excluded, which paradoxically may exclude the most refractory subgroup. I suggest to predefine stratified or subgroup analyses and clarify how prior PREEMPT exposure and response status will be accounted for analytically.

 - The rationale for the selected dose (25 U per side) is insufficiently justified. 

 The choice of a single-session intervention contrasts with real-world data suggesting cumulative effects with repeated injections. Please, provide a clearer pharmacological and anatomical rationale for dose selection and explicitly acknowledge that a negative result may reflect underdosing or insufficient exposure rather than lack of biological efficacy.

 - The primary endpoint window (weeks 5-8 post-injection) is non-standard compared with most migraine preventive trials. Justification relies almost exclusively on pilot data, without sensitivity analyses anchored to alternative windows (e.g., weeks 1-4 or 1-12). I suggest to pre-specify additional co-primary or key secondary analyses over broader time windows.

 - The protocol describes a mixed logistic regression model for a primary endpoint that is ultimately reported as a change in number of headache days, which may create conceptual and interpretative confusion. The assumed effect size (3.5 days difference) appears optimistic given the single intervention. I suggest to discuss the clinical plausibility of the assumed effect size in more depth.

 - Handling of missing diary data is permissive (“all available data”), but assumptions about missingness mechanisms are not discussed. Please, clarify how model-derived estimates translate into clinically interpretable differences in headache days and include sensitivity analyses addressing missing-not-at-random scenarios.

 - The authors acknowledge that procedure-related adverse effects may compromise blinding, but no mitigation strategy is proposed. Please, consider reporting blinding indices rather than descriptive assessments alone and discuss how partial unblinding will be handled analytically.

 - Saline injection into the SPG region may not be a fully inert placebo given mechanical or pressure-related effects. Please, clarify this aspect in more depth.

 MINOR COMMENTS

 - Clarify whether site (Oslo vs Trondheim) will be retained as a random effect regardless of statistical significance.

 - Provide more detail on training and credentialing of neurosurgeons to support reproducibility.

 - The term “quadruple-blinded” may benefit from a brief clarification early in the manuscript for non-specialist readers.

 - Consider to use a CONSORT-style flow diagram adapted for interventional procedures.

Is the study design appropriate for the research question?

Yes

Is the rationale for, and objectives of, the study clearly described?

Yes

Are sufficient details of the methods provided to allow replication by others?

Yes

Are the datasets clearly presented in a useable and accessible format?

Not applicable

Reviewer Expertise:

Clinical pharmacologist with an expertise in headache disorders

I confirm that I have read this submission and believe that I have an appropriate level of expertise to confirm that it is of an acceptable scientific standard, however I have significant reservations, as outlined above.

# Response

**Published:** 2026-01-22

**Authors:** Tore Wergeland

**Affiliations:** Department for neuromedicine, Norwegian University of Science and Technology Department of Neuromedicine and Movement Science, Trondheim, Trøndelag, Norway

Thank you for your review. We address each of you comments in the following and have revised the manuscript accordingly.

**MAJOR COMMENTS**

**- There is limited discussion of why SPG blockade should preferentially affect migraine frequency rather than cranial autonomic symptoms, which are more directly linked to SPG physiology. I suggest to expand the discussion to clarify the hypothesized mechanistic pathway linking SPG blockade to reduced migraine frequency, especially in patients already exposed to CGRP-targeting therapies.**

*Reply: We agree that the parasympathetic output via the sphenopalatine ganglion (SPG) is classically associated with certain cranial autonomic symptoms through its role in the trigemino-autonomic reflex. However, accumulating experimental and clinical evidence suggests that the SPG may also influence primary headache disorders, including treatment approaches such as SPG-stimulation in cluster headache. Examples of studies targeting the SPG are mentioned in the introduction.*

*I have added a section on limitations in the discussion, in which it is stated that 1. the current study is not designed to explore mechanisms of action, and 2. Mentions a prespecified subgroup analysis to address the fact that participants may use concomitant CGRP-targeting treatments.*

**- The population is extremely heterogeneous, including patients on CGRP monoclonal antibodies, gepants (preventive and abortive), oral preventives and PREEMPT-style onabotulinumtoxinA (historical exposure). Patients with very high prior treatment failure (> 6 drugs) are excluded, which paradoxically may exclude the most refractory subgroup. I suggest to predefine stratified or subgroup analyses and clarify how prior PREEMPT exposure and response status will be accounted for analytically.**

*Reply: The inclusion and exclusion criteria were designed to adhere to regulatory and ethical requirements and to aim to recruit a study population representative of patients who would be considered candidates for a minimally invasive SPG-targeted intervention in clinical practice.*

*The target population for this treatment is inherently heterogeneous, and the trial design intentionally reflects this by allowing continued use of preventive medications, inclusion of patients with medication overuse, and application of relatively few comorbidity-based exclusion criteria. We believe this approach enhances external validity and supports generalisability to the real-world population most likely to be considered for this intervention.*

*To address heterogeneity analytically, prespecified subgroup analyses are included in the Statistical Analysis Plan (SAP) for key baseline factors, including sex, baseline medication overuse, accompanying cranial autonomic symptoms, and concomitant use of CGRP-targeting preventive treatments. These analyses are intended to explore potential effect modification and will be clearly reported as exploratory. In addition, a prespecified adjusted mixed logistic regression sensitivity analysis is included in the SAP to assess robustness of the primary findings, adjusting for key baseline factors including site of inclusion and clinically relevant covariates.*

*Patients with documented failure of more than six preventive medications were excluded to avoid inclusion of individuals with an extremely refractory phenotype, in whom a single-session interventional approach could be less likely to demonstrate efficacy and could obscure a treatment effect. This limitation is now explicitly discussed in the manuscript.*

*Prior PREEMPT- onabotulinumtoxinA exposure and response status are prospectively recorded; however, subgroup analyses based on these variables were not prespecified in the SAP.*

**- The rationale for the selected dose (25 U per side) is insufficiently justified.**

**The choice of a single-session intervention contrasts with real-world data suggesting cumulative effects with repeated injections. Please, provide a clearer pharmacological and anatomical rationale for dose selection and explicitly acknowledge that a negative result may reflect underdosing or insufficient exposure rather than lack of biological efficacy.**

*Reply: The limitations section in the discussion now addresses dosing and possibility of a negative trial. It states: “The fixed dose of 25 IU onabotulinumtoxinA per side (total dose 50 IU) was based on results from pilot studies.¹⁸,²¹ A negative trial result may reflect underdosing or insufficient exposure, rather than absence of biological efficacy. Accordingly, future studies may need to explore repeated injections or dose-escalation strategies.”*

**- The primary endpoint window (weeks 5-8 post-injection) is non-standard compared with most migraine preventive trials. Justification relies almost exclusively on pilot data, without sensitivity analyses anchored to alternative windows (e.g., weeks 1-4 or 1-12). I suggest to pre-specify additional co-primary or key secondary analyses over broader time windows.**

*Reply: The statistical analysis plan includes analyses across multiple post-intervention periods (weeks 1–4, 5–8, and 9–12), allowing evaluation of temporal patterns of response. I have updated the statistical analysis section in the manuscript accordingly*

*Additional co-primary endpoints were not prespecified to preserve statistical rigor.*

**- The protocol describes a mixed logistic regression model for a primary endpoint that is ultimately reported as a change in number of headache days, which may create conceptual and interpretative confusion. The assumed effect size (3.5 days difference) appears optimistic given the single intervention. I suggest to discuss the clinical plausibility of the assumed effect size in more depth.**

*Reply: The assumed effect size used for the power calculation was informed by the magnitude of response observed in the pilot study in chronic migraine following a single bilateral SPG injection.*

*Importantly, the trial was designed to detect an effect size that would be considered clinically meaningful in light of the invasive nature of the procedure. A smaller difference in monthly moderate to severe headache days would, in our view, not justify an interventional approach requiring navigation-guided percutaneous injections. The manuscript has been updated accordingly.*

**- Handling of missing diary data is permissive (“all available data”), but assumptions about missingness mechanisms are not discussed. Please, clarify how model-derived estimates translate into clinically interpretable differences in headache days and include sensitivity analyses addressing missing-not-at-random scenarios.**

*Reply: The primary analysis is based on a mixed logistic regression model using all available daily diary data and is unbiased under the assumption that any missing data are missing-at-random (MAR). The publication has been updated.*

*Model-derived estimates are translated into clinically interpretable outcomes by calculating predicted marginal probabilities of moderate to severe headache per day for each 28-day period, which are subsequently converted into estimated numbers of moderate to severe headache days per month.*

*While missing-not-at-random (MNAR) mechanisms cannot be excluded in diary-based headache trials, formal MNAR sensitivity analyses are not prespecified due to their complexity when using eDiary data with 28 pre-treatment and 84 post-treatment days.  To assess robustness, prespecified sensitivity analyses include restriction to the per-protocol population (eDiary adherence over 90%).*

**- The authors acknowledge that procedure-related adverse effects may compromise blinding, but no mitigation strategy is proposed. Please, consider reporting blinding indices rather than descriptive assessments alone and discuss how partial unblinding will be handled analytically.**

*Reply: Assessment of blinding success is prespecified in the Statistical Analysis Plan (SAP). At week 12, participants are asked to indicate perceived treatment allocation (“Botox”, “Placebo”, or “Don’t know”). Blinding success will be evaluated using the blinding indices proposed by Bang et al. (2004) and James et al. (1990), calculated using the user-written Stata command blinding.*

*These indices will be used to quantify the magnitude and direction of any potential unblinding and to inform interpretation of the trial results. No adjustment of the primary efficacy analysis based on blinding status is prespecified, as post-randomisation treatment guesses may be influenced by treatment response itself.*

*Mitigation strategies includes standardised written and oral information to patients at inclusion. For the statistician, mitigation strategies includes a prespecified order of analysis, by which the blinded statistician analyses the primary endpoint before other endpoints. The statistician is granted access to AE data when all blinded analysis of efficacy endpoints are locked.*

**- Saline injection into the SPG region may not be a fully inert placebo given mechanical or pressure-related effects. Please, clarify this aspect in more depth.**

*Reply: We acknowledge that saline injection into the SPG region may not be entirely inert and could theoretically exert mechanical or pressure-related effects. However, saline was chosen as the control intervention to ensure procedural equivalence, preserve blinding, and isolate the pharmacological effect of onabotulinumtoxinA.*

*Any non-specific effects related to needle placement or injection volume would be expected to affect both groups equally and therefore bias results toward the null.*

 MINOR COMMENTS

**- Clarify whether site (Oslo vs Trondheim) will be retained as a random effect regardless of statistical significance.**

*Reply: Site will be assessed as a potential random effect prior to unblinding, as prespecified on the study SAP. The decision to retain site as a random effect will be based on their being adequate number of participants at each site, and whether the inclusion results in a better model fit.*

**- Provide more detail on training and credentialing of neurosurgeons to support reproducibility.**

*Reply: The manuscript is updated. During the course of the study, one neurosurgeon at each site performed the procedure.*

**- The term “quadruple-blinded” may benefit from a brief clarification early in the manuscript for non-specialist readers.**

*Reply: We have chosen to keep the definition as it is, and refers to the following section: This study employs rigorous allocation concealment and blinding strategies referred to as quadruple-blinding. This involves blinding of the (1) participants and (2) investigators (double blinding), in addition to blinding of the (3) statistician/outcome assessor, and (4) the pre-specified order in which the statistical analysis will be performed including preplanned tables for the analysis subject to blinded analysis.*

**- Consider to use a CONSORT-style flow diagram adapted for interventional procedures.**

*Reply: Thank you, we will consider this when preparing results from the study.*
